# Dicationic Imidazolium-Based Ionic Liquid Coatings on Zirconia Surfaces: Physico-Chemical and Biological Characterization

**DOI:** 10.3390/jfb8040050

**Published:** 2017-12-13

**Authors:** Pavan P. K. Sandhu, Izabelle M. Gindri, Danyal A. Siddiqui, Danieli C. Rodrigues

**Affiliations:** Department of Bioengineering, University of Texas at Dallas, Richardson, TX 75080, USA; pxs112930@utdallas.edu (P.P.K.S.); idg130030@utdallas.edu (I.M.G.); danyal.siddiqui@utdallas.edu (D.A.S.)

**Keywords:** ionic liquids, zirconia, dental implants, coatings, biofilm, lubrication

## Abstract

In the present work, dicationic imidazolium-based ionic liquids (ILs) were investigated as multi-functional coatings on a zirconia (ZrO_2_) surface to prevent biofilm formation and enhance the wear performance of zirconia while maintaining the material’s compatibility with host cells. ILs containing phenylalanine and methionine were synthesized and deposited on zirconia. Intermolecular interactions driving IL deposition on zirconia were studied using X-ray photoelectron spectroscopy (XPS). Anti-biofilm activity and cell compatibility were evaluated in vitro after one and seven days, and wear performance was tested using a pin-on-disk apparatus. ILs were observed to form strong hydrogen bonds with zirconia. IL containing phenylalanine formed a stable film on the surface after one and seven days in phosphate-buffered saline (PBS) and artificial saliva and showed excellent anti-biofilm properties against *Streptococcus* *salivarius* and *Streptococcus* *sanguinis*. Compatibility with gingival fibroblasts and pre-osteoblasts was maintained, and conditions for growth and differentiation were preserved. A significantly lower coefficient of friction and wear volume loss were observed for IL-coated surfaces as compared to non-coated substrates. Overall, zirconia is an emerging alternative to titanium in dental implants systems, and this study provides additional evidence of the materials’ behavior and IL coatings as a potential surface treatment technology for improvement of its properties.

## 1. Introduction

Commercially pure titanium (cpTi) has been the material of choice for oral implants during the last few decades due to their biocompatibility, corrosion resistance, and good mechanical properties, resulting in high survival rates of 90.9%–97.7% 15 years post-implantation [1,2]. However, titanium has a dark grayish color, which can sometimes be seen through the anterior peri-implant gingiva. This can jeopardize the esthetic outcome and may lead to patient dissatisfaction in some cases [3]. In addition, concerns about accumulation of metal particles in peri-implant tissues, allergies, insensitivities, corrosion, and metal intolerance have also been reported [4,5,6]. In recent years, high-strength zirconia (ZrO_2_) ceramics have garnered attention as a novel, “metal-free” implant technology and alternative materials for the components of dental implant systems. One of the attractive aspects of zirconia for oral implants is its ivory, tooth-like color. Additionally, the material’s mechanical and chemical properties, such as high fracture toughness, flexural strength and resilience, and high corrosion stability, make it a good choice for dental implant systems [7]. Despite lacking the ductility of titanium, zirconia in previous studies demonstrated a mechanical strength similar to Ti and sufficient to withstand mastication forces under simulated oral environment conditions [8,9,10]. Furthermore, recent results from clinical and in vitro studies involving zirconia demonstrated osseointegration comparable to titanium, which was also partially dependent on surface roughness and hydrophilicity of both substrates [11,12,13]. In an in vivo study, similar bone-implant contact was found on both titanium and zirconia implants after 12 weeks [14]. In a recent review study, the survival and success rates of zirconia were found to be 92% after one year of implantation [15]. Similar studies have indicated the biocompatibility and appropriate osseointegration of the material; however, these results are from short-term (less than two years) observations, and extended studies and clinical follow-up are still needed to establish long-term performance [16].

For long-term success of a dental implant system, it is necessary for the implant surface to achieve integration with soft and bone tissues, which will ensure sealing and functional integration. Furthermore, it has been discussed in the literature that soft tissue seal formation around an implant neck is critical to provide a barrier against bacterial penetration toward the bone [17]. Despite high survival rates, failures of dental implants are expected to increase, as an estimated 500,000 dental implants are placed every year in the USA alone [18,19]. Many factors can influence the survival of an implant, including implant loading procedures, microbial biofilm formation, corrosion, systemic health, and host immune–inflammatory responses [20,21,22]. Recent studies have shown that bacterial biofilm formation is one of the major factors influencing both early- as well as late-stage implant failures [22,23]. In particular, peri-implantitis associated with colonization of the surface by pathogenic bacterial strains such as *Porhyromonas gingivalis* has been implicated as a major reason for dental implant failure and estimated to affect 15%–56% of all dental implants [24]. Despite some previous studies demonstrating lower plaque accumulation and bacterial adhesion on zirconia as compared to titanium in vitro and in vivo, other studies have observed no significant differences in bacterial attachment and biofilm growth between titanium and zirconia substrates [25,26,27,28,29]. Thus, innovative methods that can provide protection of dental implant surfaces against bacterial adhesion may improve long-term performance.

Given that zirconia is a relatively new alternative material for the design of osseointegrative implant bodies or screws of two-piece dental implant systems, few studies have reported the biological and physico-chemical behavior of this material under circumstances relevant to the oral environment [16]. Likewise, technologies aimed at improving its performance for these applications are still preliminary. Therefore, the successful introduction of zirconia as a dental implant material requires a more in-depth investigation of the material’s behavior and properties, as well as investigation of novel technologies aimed at improving the response of the material within the biological environment.

Although several approaches have been proposed for the surface modification of osseointegrative implants, many of which are applicable to zirconia, there is still a need to provide multi-functionalities to an implant surface with a single surface treatment. Recently, dicationic imidazolium-based ionic liquid (IL) coatings were developed for dental implant applications. These IL coatings were functionalized to promote surfaces with antimicrobial activity, corrosion protection, and improved lubrication [30,31,32,33]. This novel series of ILs consists of a dicationic imidazolium head with an alkyl chain and an amino acid-based anionic moiety. It has been demonstrated in our previous studies that these dicationic imidazolium-based compounds, when coated on titanium surfaces, formed stable films with high adhesion strength [30]. The coatings also promoted strong antimicrobial action against bacterial species relevant to the oral environment while maintaining biocompatibility with mammalian cells comprising host soft and hard tissues [32]. An improvement in anti-corrosive and tribological properties of titanium was also observed [34]. Dicationic ILs were selected due to their lower toxicity against mammalian cells and their higher affinity to titanium surfaces in comparison to monocationic ILs [30,31,32].

Based on our previous results, two ILs with the same dicationic moiety and phenylalanine and methionine as anions were selected and investigated on zirconia substrates [30,31,32]. The goal of this study was to provide a comprehensive characterization of the surface of the material coated with dicationic imidazolium-based ILs in terms of coating stability, antimicrobial activity, cell compatibility, and lubrication behavior. It was hypothesized that the selected ILs would exhibit the same degree of multi-functional behavior on zirconia as was observed on titanium surfaces as both materials possess an oxide on their surfaces.

## 2. Results and Discussion

### 2.1. Coating Characterization

IL coatings were deposited on the surface of zirconia to investigate their adsorption and deposition profile. XPS (Physical Electronics, Inc., Chanhassen, MN, USA) and release profile studies were carried out to understand intermolecular interactions between IL and zirconia and coating stability, respectively. The hypothesis was that the carboxylate and amino groups of ILs would interact with zirconium (Zr) and oxygen (O) in the substrate. It was expected that a change in the chemical environment surrounding the constituent elements would be seen due to this interaction.

#### 2.1.1. XPS Studies

Binding energies (BEs) were used to assess the chemical interaction of ILs before and after the adsorption process on the investigated surfaces. Shifts found in these BEs that were greater than the measurement error of the equipment (±0.1 eV) were designated as a change in the chemical environment of the constituent atoms on the material’s surface.

**Figure 1:**
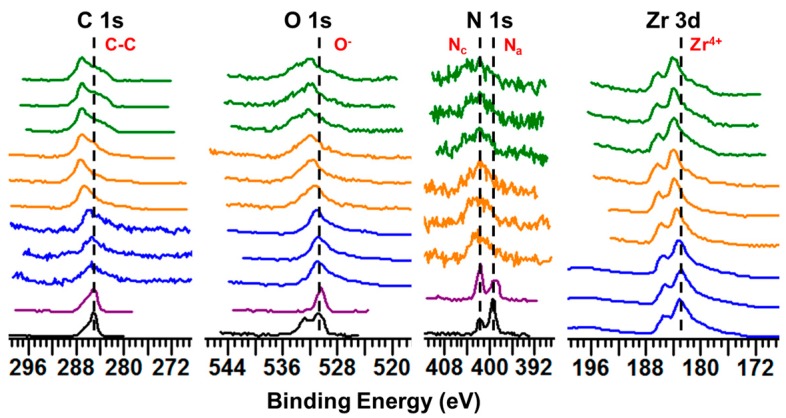
XPS spectra of C 1s, O 1s, N 1s, and Zr 3d for pure IL1 (black), pure IL2 (purple), non-coated (blue), IL1-coated (orange), and IL2-coated (green) zirconia. Dashed lines represent the BE of the primary peak components for pure IL (C 1s, O 1s, N 1s) and non-coated zirconia (Zr 3d).

According to previous studies, it was observed that the cationic moiety in the ILs can interact with negatively-charged sites, and the anionic moiety can interact with positively-charged sites [35,36]. To determine whether interaction between IL coating and zirconia surface had occurred, the BEs of elements comprising IL-coated zirconia were compared against those for pure ILs and non-coated control zirconia. The Zr 3d elemental spectra were the strongest in intensity and clearly exhibited peak splitting. For simplicity and to avoid redundancy, only the BE of the Zr 3d_5/2_ peak is discussed. XPS data for pure IL1 and IL2 are referenced based on our previous study [30]. Spectra were calibrated using the aliphatic carbon (C) 1s peak at 285.0 eV per previous studies [37,38]. The XPS spectra for the primary elements comprising IL and zirconia surface (carbon, oxygen, nitrogen, and zirconium) are shown in Figure 1.

Beginning with the C 1s spectra, aliphatic carbon was the primary peak component previously detected at 285.0 eV for pure IL1 and IL2 [30]. As for non-coated control zirconia, the peak at 285.0 eV corresponded to adventitious carbon contamination typically present on specimens exposed to ambient air. After deposition of IL1 and IL2 coatings, the aliphatic peak (representing C–C bonds in the alkyl chain, which are the primary type of C atoms in the IL structure) exhibited an increase in BE as compared to pure ILs. That is, the intensity of more polar carbon was seen to increase after deposition of ILs on zirconia surface as compared to the aliphatic carbon of pure IL. However, an aliphatic carbon peak associated with pure IL not interacting with the surface was still present but at a lower intensity. It was hypothesized that most carbon atoms in the IL interacted with the substrate, resulting in a shift of the overall peak to higher BE. This behavior was similarly observed with dicationic IL-coated titanium surfaces and monocationic IL-coated gold substrates, where a shift was seen in the aliphatic carbon peak after deposition of IL and can be attributed to the adsorption of the IL alkyl chain on the substrate [30,35].

For non-coated control zirconia, the primary O 1s peak at 530.8 ± 0.1 eV corresponded to O comprising zirconium oxide (ZrO_2_), which is in accordance with values reported in the literature for zirconia surfaces [39,40]. As for pure IL, the primary peak components at 530.4 eV and 530.7 eV were associated with oxygen in the anionic moieties of IL1 and IL2, respectively. After coating IL on zirconia, the primary peaks representing O in IL1 and IL2 increased to 531.7 ± 0.2 eV and 532.3 ± 0.0 eV, respectively. This dramatic shift to higher BE can be explained by the formation of hydrogen bonds between carboxyl groups of IL anions and Zr atoms present on zirconia. As seen in our and other previous studies, this shift to higher BE was observed to occur due to hydrogen bonds formed between O in amino acid-based moieties and titanium surfaces [30,41,42]. However, upon comparing the O 1s spectra of control and IL-coated zirconia, a shift in BE was indeed observed for O in both IL1 and IL2, suggesting that both IL coatings had interacted with the zirconia substrate.

Although the O 1s spectra suggested interaction between IL and zirconia surfaces, the N 1s spectra (Figure 1) was even more revealing due to N being unique to IL (unlike O which is shared by both IL and zirconia). Moreover, the N 1s spectra provided more information about the chemical environment surrounding IL as N present in the anionic (N_a_) and cationic (N_c_) moieties of IL exhibited separate peaks at distinct BEs. Per our previous study, pure IL1 exhibited N_a_ and N_c_ peaks at 399.1 eV and 401.8 eV, respectively [30]. After coating IL1 on zirconia, new peak components were detected at 400.2 ± 0.3 eV and 403.6 ± 0.2 eV in addition to peaks observed for pure IL. The presence of these new peaks corresponded to a shift in higher BE of both N_a_ and N_c_. The former new peak represented a shift to higher BE of N_a_ due to hydrogen bond formation between the amine of the anionic moiety and oxygen atoms present in zirconia. Likewise, a shift to higher BE of N_c_ can be attributed to this interaction as decreased charge neutralization by the IL anion is hypothesized to cause the IL cation (and thus N_c_) to become more positive [30]. However, the presence of peak components corresponding to that observed for pure IL on IL1-coated zirconia demonstrated that not all IL1 interacted with zirconia, possibly due to the IL coating being comprised of multiple layers in which IL molecules assume random orientation beyond the first layer in direct contact with the substrate [35]. Furthermore, the same trends observed for IL1 coating were also observed for IL2. That is, the presence of additional peak components detected at 399.9 ± 0.2 eV and 404.1 ± 0.3 eV on IL2-coated zirconia in addition to those observed for pure IL2 at 399.2 eV and 401.7 eV further confirmed that interaction took place between IL2 and zirconia substrate. In our previous study of IL coatings on titanium surfaces, broadening of the N 1s peak due to additional peak components was also observed and attributed to IL interaction with the underlying substrate [30]. Moreover, sulfur (S), which is unique to IL1, exhibited a large shift in BE from 163.3 eV in pure IL form to 165.7 ± 0.0 eV after coating on zirconia (Figure S1), which again emphasizes the change in chemical environment due to interaction between IL and zirconia.

In addition to the elements comprising IL, Zr comprising zirconia surfaces also exhibited increased BE values after deposition of IL on its surface. This shift in BE from 183.0 ± 0.1 eV to 183.7 ± 0.2 eV for IL1 coated zirconia and 183.9 ± 0.0 eV for IL2-coated zirconia corroborated that hydrogen bond formation occurred between oxygen in IL anion and Zr atoms on zirconia. Like titanium, zirconium in the metal oxide of ZrO_2_ can act as an acidic site and accept electrons [43]. Furthermore, the bond between Zr and O atoms comprising zirconia exhibits a greater degree of ionic character than that between Ti and O on titanium surfaces, which can further drive the interaction of IL with zirconia surfaces [43]. Overall, the results of XPS analysis indicated that interaction between both IL1 and IL2 with zirconia surfaces occurred via strong hydrogen bonds formed between carboxylate and amino groups of ILs and elements comprising the substrate.

#### 2.1.2. Release Profile

In this study, IL coating release in aqueous media was investigated after one and seven days of immersion to evaluate the stability of the IL coating during the time frame in which initial healing occurs post-implantation. Phosphate-buffered saline (PBS) and artificial saliva were chosen to simulate relevant physiological conditions.

**Figure 2:**
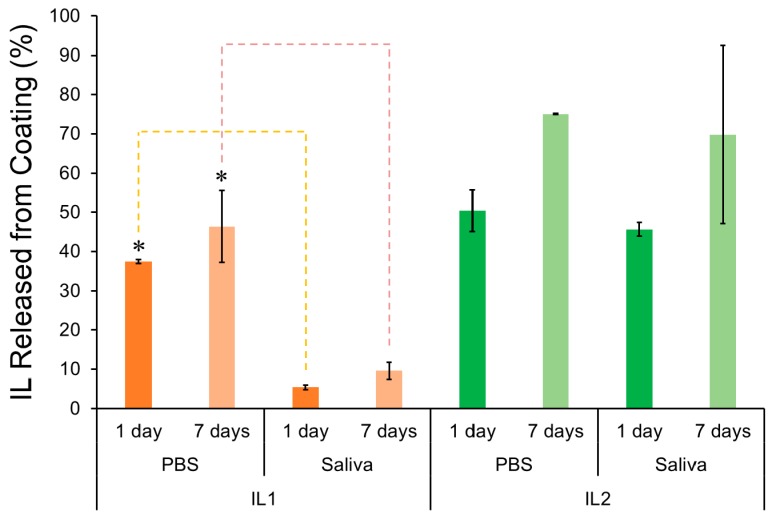
Percentage of IL1 and IL2 released from coated specimens immersed in PBS and saliva. * Significantly different (*p* < 0.05). Note: The error bars are standard deviations from the mean calculated from triplicate results, and dashed lines indicate groups between which significant difference was observed.

Figure 2 shows the percentage of IL released from IL-coated specimens immersed in PBS and saliva. After one day of immersion in both media, lower IL release was observed for IL1-coated zirconia specimens (33.9% ± 0.5% and 4.9% ± 0.5% in PBS and saliva, respectively) in comparison to IL2-coated zirconia (43.2% ± 4.6% and 39.2% ± 1.5%, in PBS and saliva, respectively). Although both IL1 and IL2 contain amino acids (phenylalanine and methionine, respectively) as anionic moieties, the presence of an aromatic ring in phenylalanine contributes to a more hydrophobic compound as compared to methionine, which will not get as easily solvated [44]. As a result, it can be expected that more IL molecules of IL1 coating would remain on the surface of zirconia and not diffuse into the immersion media relative to IL2. In addition to structural differences in IL composition, the difference in pH of the immersion media (saliva with a pH of 4.9 and PBS with a pH of 7.4) was observed to significantly affect the release profile of ILs from zirconia. A significantly lower release was seen in saliva as compared to PBS for IL1-coated specimens at both time points investigated (one day and seven days) (*p* < 0.05), which was also previously observed for IL1 coatings on titanium surfaces [32]. Similarly, IL2 coating was observed to have slightly lower average release after both one and seven days of immersion in saliva as compared to PBS, though this difference was not significant (*p* > 0.05). 

After seven days, the percentage of IL1 released in PBS and saliva relative to the initial amount of IL coated was 41.9% ± 8.3% and 8.7% ± 1.9%, respectively, and for IL2, it was 64.4% ± 8.7% and 59.9% ± 19.5%, respectively. Even after seven days of immersion in PBS and saliva, the results showed that IL release was not significantly (*p* > 0.05) increased when compared to the results obtained for one day of immersion in both media. These results corroborated trends seen in our previous study on IL-coated titanium surfaces [32]. However, unlike our previous immersion study, IL release from IL2-coated zirconia was not found to be significantly lower in saliva than in PBS after one and seven days of immersion as was seen for titanium surfaces (*p* > 0.05), suggesting that IL2 had a lower affinity for the zirconia surface than for titanium ones.

Coating stability is an essential requirement for the maintenance of surface functionalization during the initial healing period post-implantation. Studies have shown that matrix-rich regeneration tissue starts to develop between the bone and implant surface during the first week of implantation [14]. Therefore, considering the initial healing period occurs within the first two weeks post-implantation, IL coatings should remain intact for at least 1–2 weeks. Thus, the results observed for IL-coated zirconia were promising since IL was found to adhere to zirconia surfaces even after seven days of immersion in PBS and saliva.

### 2.2. Mammalian and Bacterial Cell Activity

The goal of this part of the study was to assess the fundamental behavior of mammalian host and bacteria cells on control zirconia and IL-coated zirconia surfaces in terms of growth and differentiation for mammalian cells and concentration of bacteria for bacterial cells.

#### 2.2.1. Mammalian Cell Activity

**Figure 3:**
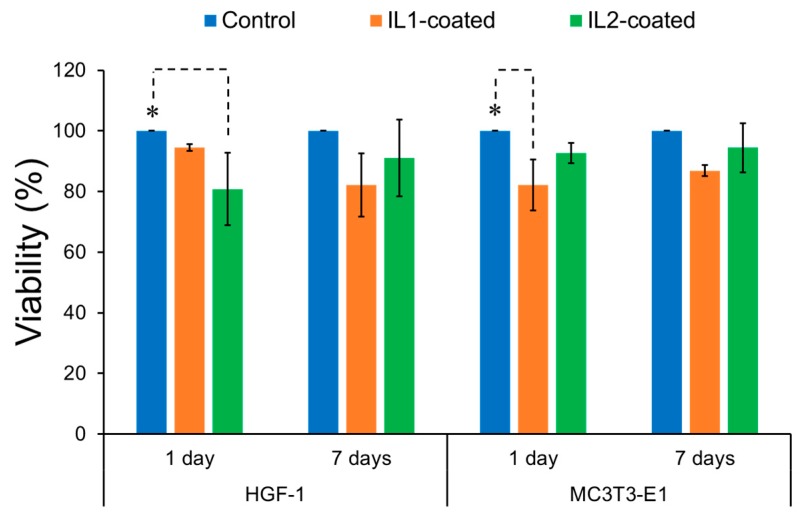
Cell viability (%) of IL-coated zirconia relative to non-coated control for HGF-1 fibroblasts and MC3T3-E1 pre-osteoblasts after one and seven days. * Significantly different (*p* < 0.05). Note: The error bars are standard deviations from the mean calculated from triplicate results, and dashed lines indicate groups between which significant difference was observed.

To better understand the cytocompatibility of IL-coated zirconia, cell viability was evaluated with human gingival fibroblasts (HGF-1) and mouse pre-osteoblasts (MC3T3-E1) after exposure to IL-coated and non-coated control zirconia specimens, and the results are summarized in Figure 3. For HGF-1 cells, IL1-coated zirconia showed similar viability values (94.6%) as control zirconia (100%) after one day (*p* > 0.05). In contrast, IL2 resulted in significant reduction in cell viability (80.8%) when compared to control zirconia (*p* < 0.05). After seven days, both IL1 (82.2%) and IL2-coated (90.1%) zirconia specimens resulted in lower cell viability than control zirconia; however, these viability values were not statistically different than the control (*p* > 0.05). For pre-osteoblast cells, IL2-coated zirconia resulted in similar cell viability (92.7%) as control zirconia (100%) after one day, whereas IL1-coated zirconia displayed significantly lower viability values (82.2%) relative to control zirconia (*p* < 0.05). After seven days, viability values obtained for IL1-coated (87%) and IL2-coated (94.4%) specimens were similar to control zirconia and no significant differences were observed between them (*p* > 0.05).

Per ISO 10993-5:2009 standard, which assesses cell viability on the surface of biomaterials, a more than 30% of reduction in cell viability is considered a cytotoxic effect. Since cell viabilities obtained for all IL-coated specimens were above 80% across both cell lines, the IL coatings can be considered non-cytotoxic. Furthermore, the trends observed in these results correlated with the release profile of IL1-coated and IL2-coated zirconia in PBS as discussed previously. That is, IL coating was observed to have lower average viability for fibroblasts and pre-osteoblasts after one day of exposure relative to non-coated specimens. However, cell viability in general was observed to increase and recover after seven days. It can be assumed that since a high concentration of IL1 and IL2 was released after the first day of immersion in this medium, lower viability would be expected for IL-coated zirconia after one day. However, IL release did not significantly increase between one and seven days based on the release profile, implying that IL coating became stable after one day and therefore could not release substantially more IL. Furthermore, cell culture medium was changed every 48 h, thereby removing the initial amount of IL released. As a result, mammalian cells would be exposed to lower amounts of IL in the surrounding media, thereby lessening the cytotoxic effect of ILs and allowing for increased cell viability by the seven-day time point.

In our previous study, IL1-coated titanium specimens showed more than 70% cell viability for both MC3T3-E1 pre-osteoblasts and HGF-1 fibroblasts after seven days, which was similar to the results found for IL1-coated zirconia [32]. Interestingly, cell viability for IL2-coated zirconia for both cell lines was found to be higher than IL2-coated titanium specimens after seven days. This behavior may have been observed for IL2 due to a lower amount of IL being deposited on each zirconia specimen (1.1 µmol) than on each titanium specimen (2 µmol) [32]. In the previous study, IL2 coating in general was observed to have greater cytotoxicity relative to IL1 due to having methionine as its anionic moiety [32]. However, the use of lower concentrations for IL2-coated zirconia in this study in comparison to our previous work may have dampened this effect, resulting in cell viability values similar to that for IL1 coating and thus being suitable for in vivo applications.

**Figure 4:**
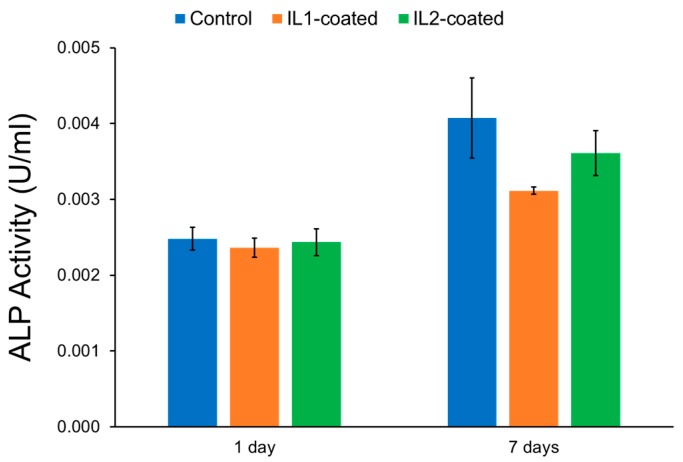
ALP activity of MC3T3-E1 cells after exposure to control zirconia, IL1-coated, and IL2-coated zirconia specimens after one and seven days. Note: The error bars are standard deviations from the mean calculated from triplicate results.

**Figure 5:**
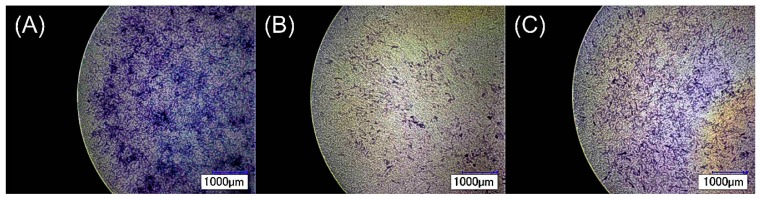
ALP staining (purple coloration) of differentiated MC3T3-E1 cells attached on the surface of (**A**) non-coated control; (**B**) IL1-coated; and (**C**) IL2-coated zirconia after seven days.

The differentiation of pre-osteoblasts on IL-coated and non-coated control zirconia were determined by their alkaline phosphatase (ALP) activity, and the results are summarized in Figure 4. Differentiation of pre-osteoblasts into osteoblasts correlates to a high ALP activity in cells. ALP is a common biochemical marker and is used to identify the differentiation of pre-osteoblasts to osteoblasts in vitro. Increase in the levels of ALP activity is often related to the initiation of bone mineralization [32]. It was found that although progenitor pre-osteoblast cells exposed to both IL-coated zirconia exhibited lower viabilities in relation to control, the presence of IL on the surface did not affect their differentiation into osteoblasts. The ALP values of IL-coated and non-coated zirconia were similar to each other both after one and seven days (*p* > 0.05). However, due to the low ALP values, this data are not relevant. The ALP staining results for the surfaces of control, IL1-coated, and IL2-coated zirconia after seven days are shown in Figure 5. It was observed that pre-osteoblast cells were able to attach and differentiate into osteoblasts on the surface of IL-coated zirconia specimens.

#### 2.2.2. Bacterial Cell Activity

**Figure 6:**
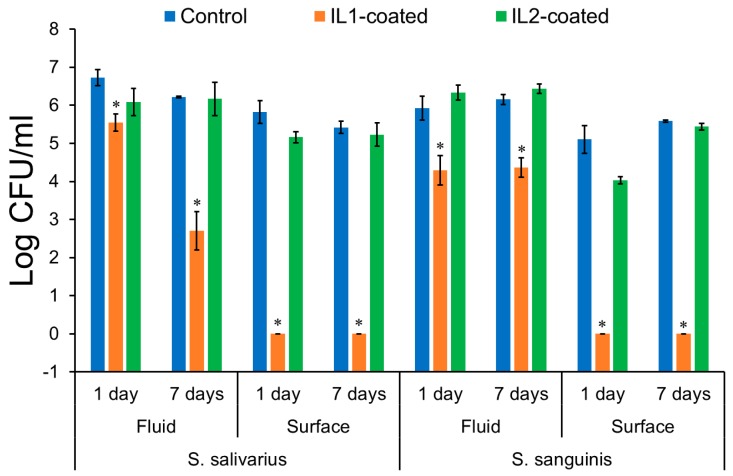
Log CFU/mL in fluid and on surface of control and IL-coated zirconia after one day and seven days of exposure to *S. salivarius* or *S. sanguinis*. * Significantly different than control within group (*p* < 0.05). Note: The error bars are standard deviations from the mean calculated from triplicate results.

Anti-biofilm activity of IL1-coated and IL2-coated zirconia was tested against *S. salivarius* and *S. sanguinis* for one and seven days in culture broth containing brain heart infusion (BHI) and saliva, and the results are summarized in Figure 6. IL1-coated zirconia specimens produced a significantly reduced bacterial count (log colony-forming units per ml (CFU/mL)) in the immersion fluid of both *S. salivarius* (5.5 ± 0.2) and *S. sanguinis* (4.3 ± 0.4) as compared to control zirconia (6.7 ± 0.2 and 5.9 ± 0.3, respectively) after one day (*p* < 0.05). The surfaces of specimens coated with IL1 were found to be almost devoid of bacteria (below the limit of detection) for both *S. salivarius* (0.0 ± 0.0) and *S. sanguinis* (0.0 ± 0.0), with significant reduction in relation to control zirconia (5.8 ± 0.3 and 5.1 ± 0.4, respectively) (*p* < 0.05). However, IL2-coated zirconia specimens did not exhibit anti-biofilm activity and had similar bacterial load as control zirconia. Bacterial count (log CFU/mL) in immersion fluid of IL2-coated specimens for *S. salivarius* (6.1 ± 0.4) and *S. sanguinis* (6.3 ± 0.2) was found to be similar to control zirconia (6.7 ± 0.2 and 5.9 ± 0.3, respectively) (*p* > 0.05). Similarly, no significant difference was found between the *S. salivarius* (5.2 ± 0.2) and *S. sanguinis* (4.0 ± 0.1) loads on IL2-coated zirconia in relation to control zirconia (5.8 ± 0.3 and 5.1 ± 0.3, respectively) (*p* > 0.05). These results correlated with the release profile for IL coating previously discussed. For IL1, the amount of coating released was only 4.9% ± 0.5% after 1 day of immersion in saliva. However, this still led to the decrease in CFU/mL measured in the immersion fluid and maintained no bacteria (below detection limit) on the zirconia substrate. In contrast, this behavior was not observed for IL2 coating despite its release profile demonstrating that IL2 was present on both surface and in immersion media.

After seven days of immersion, the anti-biofilm activity of IL1-coated zirconia was still retained, which resulted in significantly lower count of bacteria (log CFU/mL) in the immersion fluid of *S. salivarius* (2.7 ± 0.5) and *S. sanguinis* (4.4 ± 0.3) as compared to control zirconia (6.2 ± 0.0 and 6.1 ± 0.1, respectively) (*p* < 0.05). Again, the surface of IL1-coated specimens showed absence of bacterial colonies and significantly lower bacteria count for *S. salivarius* (0.0 ± 0.0) and *S. sanguinis* (0.0 ± 0.0) after seven days in relation to control zirconia (5.4 ± 0.16 and 5.6 ± 0.0, respectively) (*p* < 0.05). Correlating these results with the coating release profile, 8.7% ± 1.9% of IL1 was released in saliva from zirconia surface after seven days. Since the amount released was still relatively low, it helped to maintain the antimicrobial activity both in fluid and on the surface. However, like the one-day results, IL2-coated zirconia specimens exhibited a similar number of bacterial colonies as the control zirconia after seven days (*p* > 0.05). This behavior has been seen in our previous work, and it is assumed that the absence of antimicrobial activity of IL2 may be attributed to the formation of crystals between the IL molecules and compounds found in the artificial saliva. The crystallization was suggested to inactivate the IL molecules and therefore inhibit the antimicrobial activity of IL2 [32].

Despite few studies suggesting lower bacterial load on zirconia surfaces in physiological conditions relative to titanium, a number of other studies have demonstrated similar bacterial adherence and biofilm formation on titanium and zirconia surfaces [28,45]. Therefore, IL coatings selected in this study are relevant as they can provide anti-biofilm activity to the implant surface during the initial healing period. Zirconia specimens coated with IL1 demonstrated strong antimicrobial activity after one and seven days, whereas no such activity was observed for IL2-coated zirconia. These results demonstrate that IL1 coating may constitute an effective surface treatment to reduce biofilm formation on zirconia surfaces.

### 2.3. Tribological Behavior

**Figure 7:**
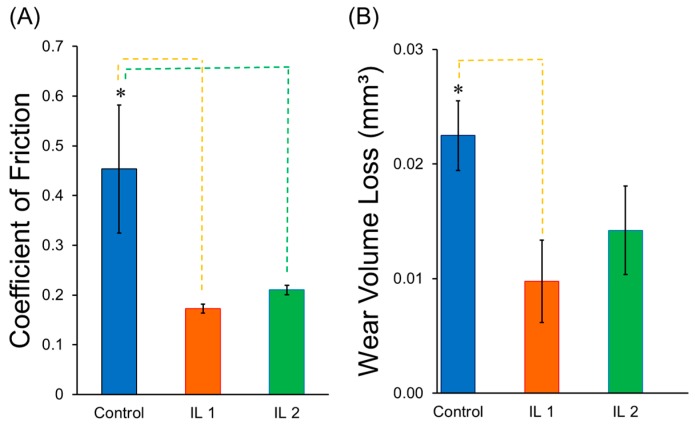
(**A**) Coefficient of friction (COF) and (**B**) total wear volume loss of control and IL-coated zirconia. * Significantly different (*p* < 0.05). Note: The error bars are standard deviations from the mean calculated from triplicate results, and dashed lines indicate groups between which significant difference was observed.

To investigate the effect of the two ILs on the tribological behavior of zirconia, wear tests were performed using experimental conditions that represented worst-case scenarios for the implant experiencing stresses during insertion, mastication, and micromotion [46,47]. Coefficient of friction and wear volume loss were calculated, and the results are shown in Figure 7.

IL coatings were found to be stable throughout testing and able to reduce friction between the two surfaces in contact (Figure S2). A significant sustained decrease in the coefficient of friction values was observed for IL1-coated and IL2-coated zirconia as compared to non-coated control zirconia (*p* < 0.05). Wear volume loss for IL1-coated zirconia was significantly lower than control zirconia specimens (*p* < 0.05). IL2-coated zirconia also exhibited lower wear volume loss as compared to the control. However, this reduction was not significantly different (*p* > 0.05).

The results showed that both IL compositions resulted in a significant reduction in COF compared to control specimens. This reduction can be attributed to the presence of long alkyl chains in both IL structures. As discussed in the literature, longer alkyl chains help to form more stable layers on titanium surfaces, which in turn lead to lower wear loss [48,49]. Also, the hydrophobicity of anions plays an important role in determining the lubrication performance of ILs. It has been reported that an increase in hydrophobicity results in lower COF and wear volume loss values [48]. Therefore, it was expected that the anion of IL1 (phenylalanine), which is more hydrophobic than IL2 (methionine), would result in slightly better lubrication performance as compared to IL2 for zirconia surfaces as was previously observed for titanium substrates [34].

Regarding lubricant film stability, ILs formed a stable layer on zirconia surfaces. COF values for IL1-coated and IL2-coated zirconia were found to be stable throughout test indicating hydrodynamic lubrication between the stainless steel ball and zirconia specimens (Figure S2). As seen previously in the coating release profile study, selected ILs were found to be more stable in saliva and had a lower release in saliva as compared to PBS. Thus, the protective layer formed by IL was maintained in the solution throughout the testing, and stable COF values were obtained for both IL-coated zirconia.

**Figure 8:**
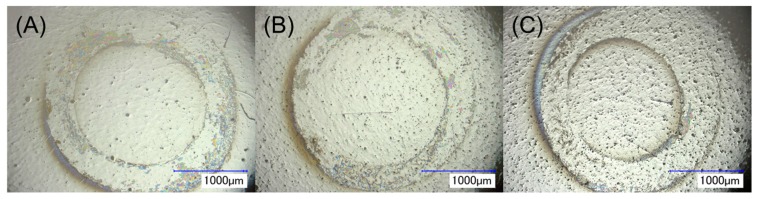
Wear scars generated on (**A**) control, (**B**) IL1-coated, and (**C**) IL2-coated zirconia after tribological testing under an applied axial load of 10 N.

As seen in Figure 8, wear scars formed on control zirconia surfaces were generally found to be nearly to completely circular and prominent for all three specimens. On IL-coated specimens, the wear scars were not as circular, and in particular, the wear track width for IL1-coated zirconia was found to be comparatively thinner than control zirconia. This was due to slipping occurring on the surface during wear testing, demonstrating lubricant activity provided by this IL. Overall, IL1-coated specimens were seen to have significantly less wear generation due to lower wear volume loss than control zirconia specimens (*p* < 0.05). Moreover, the total wear volume loss for IL-coated specimens can mainly be attributed to wear generation from the stainless steel ball and not the zirconia specimen. That is, the appearance of relative large wear track width observed for the wear scars on zirconia was due to superficial scratching by the stainless steel ball as minimal indentation was visible on its surface. Furthermore, the presence of the regions within the wear scar that appeared to be pristine and similar to regions of the surface adjacent to the wear scar indicated that minimal damage had occurred on the zirconia surface. As stainless steel is less wear-resistant than zirconia, the circular contact area between the ball and specimen became increasingly larger, allowing for more superficial damage on the zirconia surface. In the context of dental implant systems, frictional forces applied during insertion of an implant, mechanical stresses, and micromotion are factors contributing to early implant loss by hampering the surface stability of the implant during the initial phases after implantation [47]. Lubrication provided by ILs on implant surfaces can help minimize such frictional and wear effects, which may facilitate implant insertion.

Despite the multi-functional behavior imparted by IL1 coating on zirconia surface, some limitations of the present study need to be addressed. Although IL1 demonstrated coating stability due to a stable release profile being observed in PBS and artificial saliva after seven days, this testing was performed under stagnant conditions and with the immersion media not being replaced periodically, thereby possibly mitigating further release of the IL from the coating due to reaching dynamic equilibrium. During evaluation of mammalian cell activity, media was changed every 48 h, removing IL initially released into the cell culture medium. This reduction in exposure to IL, although necessary to assess recovery of pre-osteoblasts and gingival fibroblasts by the seven-day time point, did not allow for evaluation of growth under continuous seven-day exposure to IL. Future studies will overcome these limitations by evaluating the IL release profile, antimicrobial activity, and mammalian cell growth under fluid-flow conditions to better mimic fluid movement in the oral cavity while allowing for continuous release and exposure to IL.

## 3. Materials and Methods

### 3.1. Sample Preparation

**Figure 9:**
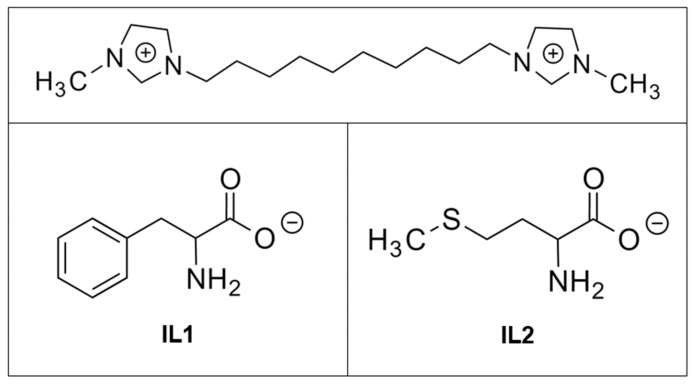
Chemical structure of ILs investigated in this work.

1,10-Bis(3-methylimidazolium-1-yl)decane diphenylalanine (IL1) and 1,10-bis(3-methylimidazolium-1-yl)decane dimethionine (IL2) were synthesized following protocols discussed in our previous studies [31,32]. Both ILs were characterized using ^1^H nuclear magnetic resonance spectroscopy (NMR) (DPX 400, Bruker, Billerica, MA, USA), and the data found were in accordance with the literature [31]. The chemical structures of IL1 and IL2 are depicted in Figure 9.

Wear-resistant zirconia (ZrO_2_) ceramic rods partially stabilized with 3% magnesium oxide (MgO) were obtained (McMaster-Carr, Elmhurst, IL, USA). The rods were cut into disks with 0.95 cm diameter and 0.45 cm height using a precision cutter equipped with a diamond blade (IsoMet 1000, Buehler, Lake Bluff, IL, USA) and were subsequently mounted for polishing using epoxy resin. Mounted specimens were polished using an automated polishing head (FEMTO 1100, Pace Technologies, Tucson, AZ, USA) and plate (NANO 1000T, Pace Technologies, Tucson, AZ, USA) by applying a set force of 90 psi at a speed of 200 rpm for 5 min per step, excluding the last step, which ran at 100 rpm. The polishing procedure included 30 µm polycrystalline diamond solution, 6 µm polycrystalline diamond solution, 1 µm polycrystalline diamond solution, and 40% colloidal silica solution in sequential steps. The polished specimens were then removed from the resin molds and cleaned by ultrasonicating in acetone, deionized water, and ethanol for 15 min each before drying in an oven at 60 °C for 48 h. To coat IL on the zirconia substrates, 0.011 mM ethanolic solutions of IL1 and IL2 were prepared. Five microliters of the coating solution was pipetted onto specimens 10 times at an interval of 15 min. The coated specimens were then dried in an oven at 60 °C for 48 h. Three coated and three non-coated samples were tested in each of the following experiments.

### 3.2. Morphological and Physico-Chemical Characterization of IL-Coated Zirconia

X-ray photoelectron spectroscopy (Physical Electronics, Inc., Chanhassen, MN, USA) was used to analyze the surface elemental composition and interaction between IL coating and zirconia surface, and ultraviolet-visible (UV-Vis) spectroscopy (Thermo Scientific, Waltham, MA, USA) was used to study the IL release from the coated specimens.

#### 3.2.1. X-ray Photoelectron Spectroscopy

One non-coated control and IL-coated zirconia specimen for each IL were analyzed on three different areas per specimen using X-ray photoelectron spectroscopy (XPS, PHI 5000 Versa Probe II, Physical Electronics Inc., Chanhassen, MN, USA) X-ray Photoelectron Spectrometer, (Physical Electronics, Inc., Chanhassen, MN, USA). A monochromatic Al Kα source of 1486.6 eV was used, and the measurements were taken at an angle of 45° with respect to the surface of the specimen. The pressure in the analysis chamber was kept below 10^−8^ torr, and the survey spectra were obtained using a pass energy of 187.850 eV and 0.8 eV step size. The high-resolution spectra were acquired using a pass energy of 23.5 eV and step size of 0.2 eV.

#### 3.2.2. Release Profile

To study IL coating release into the immersion media, non-coated, IL1-coated, and IL2-coated zirconia (*n* = 3) were each immersed in 1 mL of 1X phosphate-buffered saline (PBS, Fisher Scientific, Hampton, NH, USA) or Fusayama/Meyer artificial saliva (Pickering Laboratories, Mountain View, CA, USA), separately in 24-well plates. The plates were maintained at 37 °C for one day and seven days. After one and seven days, absorbance was recorded from the 2 µL aliquots obtained from each well using an ultraviolet-visible (UV-Vis) spectrophotometer (NanoDrop 2000, Thermo Scientific, Waltham, MA, USA). The corresponding IL concentration in the immersion media was determined using previously established calibration curves for IL1 and IL2 in PBS and artificial saliva [32].

### 3.3. Anti-Biofilm Activity of IL-Coated Zirconia

Two *Streptococcus* species, *Streptococcus sanguinis* (ATCC 10556, American Type Culture Collection, Manassas, VA, USA) and *Streptococcus salivarius* (ATCC 13419, American Type Culture Collection, Manassas, VA, USA), were chosen to evaluate the anti-biofilm activity of IL-coated zirconia specimens against early bacterial colonizers. The bacterial strains were streaked on BHI agar plates and then incubated in microaerophilic atmosphere at 37 °C. The bacterial inoculation was incubated and then diluted with 1.9:0.1 ratio of artificial saliva/BHI with a concentration of 10^5^ colony-forming units per ml (CFU/mL). For the one-day test, control and IL-coated zirconia specimens (*n* = 3) were immersed for 24 h in cell-density-normalized bacterial culture. Thereafter, the specimens were taken out and washed three times gently with buffer to remove non-adherent bacterial cells. The specimens were then vortexed vigorously in buffer, and the surface of the specimens was scraped with the help of a sterile spatula to remove any bacteria attached to the surface. The bacterial growth in immersion media and on specimen surface was then quantified in CFU/mL by plating serial dilutions of the immersion fluid and buffer solution on BHI agar plates. To evaluate the long-term antimicrobial activity of IL coatings, non-coated and IL-coated zirconia specimens (*n* = 3) were immersed in artificial saliva/BHI medium for six days. On the seventh day, bacterial inoculation, removal of bacterial biofilm, and quantification of bacterial growth in the immersion and specimen surface were performed as described above for one-day testing.

### 3.4. Evaluation of Mammalian Cell Behavior on IL-Coated Zirconia

Human gingival fibroblasts (HGF-1), murine pre-osteoblast cells (MC3T3-E1), Dulbecco’s modified eagle medium, alpha minimum essential medium, and trypsin were obtained from the American Type Culture Collection. Agar and BHI were purchased from Fisher Scientific.

#### 3.4.1. Cell Culture

To understand and evaluate host cell integration on IL-coated zirconia, cell culture tests were performed in vitro using six-well plates for one and seven days. Human gingival fibroblasts (HGF-1) and murine pre-osteoblast cells (MC3T3-E1) of the fifth to seventh passages were used for this study. The cells were cultured in Dulbecco’s modified eagle medium with 10% fetal bovine serum and alpha minimum essential medium with 10% bovine serum, respectively, at 37 °C and 5% CO_2_ in a humid environment. Control and IL-coated zirconia (*n* = 3) were then placed individually in six-well plates with seeding density of 350,000 cells per well for one-day tests and 100,000 cells per well for seven-day tests. These cell densities were chosen to ensure the full confluency of cells and hence detect differences in cell proliferation at specific time points of the study [31,32]. The culture medium was changed every 48 h during the seven-day tests.

#### 3.4.2. Cell Viability

Cell viability for fibroblasts and osteoblasts after being exposed to control zirconia and IL-coated zirconia for one and seven days was evaluated using MTT (3-(4,5-dimethylthiazol-2-yl)-2,5-diphenyltetrazolium bromide) assay, as described in detail in our previous study [32]. Cells adhering to the specimen and well surface were transferred to 96-well plates and subsequently exposed to MTT reagent and then detergent. Cell viability was quantified by measuring the absorbance values at 570 nm after production of blue formazan by viable cells upon exposure to MTT. The cell viability percentages were then calculated with respect to the wells with control zirconia, after subtracting the blank value from each well.

#### 3.4.3. ALP Activity

ALP expression was evaluated in two ways, as described in detail in our previous study [32]. A colorimetric assay (Abcam, Cambridge, MA, USA) was used to quantify the amount of ALP produced by osteoblasts in contact with control and IL-coated zirconia. Also, differentiated cells were stained with 5-bromo-4-chloro-3-indolyl phosphate/nitro blue tetrazolium (MyBioSource, San Diego, CA, USA) to observe their morphology and attachment using optical microscopy (VHX-2000 Digital Microscope, Keyence, Osaka, Japan).

### 3.5. Investigation of the Tribological Behavior of IL-Coated Zirconia

The wear behavior of IL-coated zirconia in comparison to non-coated specimens was evaluated in artificial saliva maintained at 37 °C. A continuous sliding motion using a modified pin-on-disk apparatus mounted on a hybrid rheometer (DHR-3, TA Instruments, New Castle, DE, USA) was used to perform wear testing. The method employed followed procedures described in ASTM G99-05 and ASTM G133-05.

A stainless steel ball (12.7 mm) was fixed in a modified ball specimen holder consisting of a semi-spherical fluid compartment as shown in Figure S3. Two milliliters of artificial saliva were then added into the fluid compartment, which completely submerged the stainless steel ball. The radius of the circular wear scar on the zirconia specimens was maintained at 1.25 mm. Then 10 N of axial load was applied on mounted specimens, corresponding to a maximum elastic contact stress of 839 MPa. The relative speed between the contact point of the ball and specimens was kept constant at 0.05 m/s for a total sliding distance of 50 m. The frictional and axial force values were measured at a 1 Hz sampling rate and used to calculate the coefficient of friction.

After wear testing, the specimens were cleaned with acetone to remove wear debris generated during testing and observed with optical microscopy. The average width of wear scars was measured from six different points located on the circular wear scar using optical microscopy. The wear volume loss was then calculated using Equations (1)–(3) below defined in ASTM G99-05, which are applicable to a disk and spherical pin sliding against each other under elastic deformation conditions.

(1) $$\mathrm{Disk}\;\mathrm{Volume}\;\mathrm{Loss}=2\pi R\left[r^{2}\sin^{-1}\left(\frac{d}{2r}\right)-\left(\frac{d}{4}\right){\left(4r^{2}-d^{2}\right)}^{\frac{1}{2}}\right]$$

(2) $$\mathrm{Pin}\;\mathrm{Volume}\;\mathrm{Loss}=(\frac{\pi h}{6})(\frac{3d^{2}}{4}+h^{2})$$

(3) $$h=r-{\left(r^{2}-\frac{d^{2}}{4}\right)}^{\frac{1}{4}}$$

where,

R = Radius of the wear track formed on zirconia specimen.
r = Diameter of the spherical pin.
d = Width of the wear track on zirconia specimen or the diameter of the wear scar on the ball.
h = Wear depth of the flat wear scar on the ball.

### 3.6. Statistical Analysis

Statistical significance between non-coated and IL-coated zirconia was determined for parameters used to assess coating stability, mammalian cell cytotoxicity, anti-microbial activity, and lubricant behavior. One-way analysis of variance (ANOVA) and post-hoc Tukey tests were performed using Origin Pro 8 software (OriginLab Corporation, Northampton, MA, USA), and the between-group means were deemed significantly different for *p*-values ≤ 0.05.

## 4. Conclusions

In conclusion, dicationic imidazolium-based IL coatings containing phenylalanine (IL1) and methionine (IL2) were found to interact with zirconia surfaces based on XPS analysis, which resulted in a shift to higher BE values for all elements comprising IL and zirconia. Similarly, both IL1 and IL2 coatings were found to be stable after one and seven days of immersion in either PBS or artificial saliva. However, IL1 coating demonstrated greater film stability in saliva as compared to IL2. Both IL1 and IL2 coatings on zirconia were deemed to be non-cytotoxic to both HGF-1 fibroblasts and MC3T3-E1 pre-osteoblasts after one and seven days of exposure. On the other hand, only IL1 coating demonstrated anti-biofilm activity against early-colonizing *S. sanguinis* and *S. salivarius* on both the surface of zirconia and the surrounding immersion media, while IL2 coating exhibited bacterial loads similar to those of non-coated control zirconia. Evaluation of the anti-biofilm mechanism of these IL-coatings as well as the interaction between IL and zirconia surface after long-term bacterial cell incubation will be investigated in future studies. As a lubricant, both IL1 and IL2 demonstrated excellent wear performance for zirconia substrates, resulting in sustained decreases in coefficient of friction and wear volume loss generated. Within the limitations of this study, IL1 demonstrated the best performance as a multi-functional coating for zirconia surfaces by providing antimicrobial activity and lubrication as well as maintaining a suitable environment for host cell attachment, proliferation, and differentiation.

## Supplementary Materials

The following are available online at www.mdpi.com/2079-4983/8/4/50/s1. Figure S1 is the elemental S 2p spectra for pure IL2 (purple) and IL2-coated zirconia (green). Figure S2 is coefficient of friction of control and IL-coated zirconia under a load of 10 N over a sliding distance of 50 m. Figure S3 is the modified pin-on-disk apparatus used for this study. A semi-circular fluid holder containing saliva completely submerses the stainless steel ball while the zirconia specimen is mounted to the upper fixture.

## References

[1] Chehroudi B., Gould T.R.L., Brunette D.M. (1992). The Role of Connective Tissue in Inhibiting Epithelial Downgrowth on Titanium-Coated Percutaneous Implants. J. Biomed. Mater. Res..

[2] Moraschini V., Poubel L.A.C., Ferreira V.F., Barboza E.S.P. (2015). Evaluation of Survival and Success Rates of Dental Implants Reported in Longitudinal Studies with a Follow-Up Period of at Least 10 Years: A Systematic Review. Int. J. Oral Maxillofac. Surg..

[3] Heydecke G., Kohal R., Gläser R. (1999). Optimal Esthetics in Single-Tooth Replacement with the Re-Implant System: A Case Report. Int. J. Prosthodont..

[4] Sicilia A., Cuesta S., Coma G., Arregui I., Guisasola C., Ruiz E., Maestro A. (2008). Titanium Allergy in Dental Implant Patients: A Clinical Study on 1500 Consecutive Patients. Clin. Oral Implants Res..

[5] Goutam M., Giriyapura C., Mishra S.K., Gupta S. (2014). Titanium Allergy: A Literature Review. Indian J. Dermatol..

[6] Lalor P.A., Revell P.A., Gray A.B., Wright S., Railton G.T., Freeman M.A. (1991). Sensitivity to Titanium. A Cause of Implant Failure?. J. Bone Jt. Surg. Br..

[7] Piconi C., Maccauro G. (1999). Zirconia as a Ceramic Biomaterial. Biomaterials.

[8] Andreiotelli M., Kohal R.-J. (2009). Fracture Strength of Zirconia Implants after Artificial Aging. Clin. Implant Dent. Relat. Res..

[9] Kohal R.J., Wolkewitz M., Tsakona A. (2011). The Effects of Cyclic Loading and Preparation on the Fracture Strength of Zirconium-Dioxide Implants: An In Vitro Investigation. Clin. Oral Implants Res..

[10] Spies B.C., Nold J., Vach K., Kohal R.-J. (2016). Two-Piece Zirconia Oral Implants Withstand Masticatory Loads: An Investigation in the Artificial Mouth. J. Mech. Behav. Biomed. Mater..

[11] Mihatovic I., Golubovic V., Becker J., Schwarz F. (2017). Bone Tissue Response to Experimental Zirconia Implants. Clin. Oral Investig..

[12] Zhao B., Van Der Mei H.C., Subbiahdoss G., De Vries J., Rustema-Abbing M., Kuijer R., Busscher H.J., Ren Y. (2014). Soft Tissue Integration Versus Early Biofilm Formation on Different Dental Implant Materials. Dent. Mater..

[13] Zhao B., van der Mei H.C., Rustema-Abbing M., Busscher H.J., Ren Y. (2015). Osteoblast Integration of Dental Implant Materials after Challenge by Sub-Gingival Pathogens: A Co-Culture Study In Vitro. Int. J. Oral Sci..

[14] Depprich R., Zipprich H., Ommerborn M., Naujoks C., Wiesmann H.-P., Kiattavorncharoen S., Lauer H.-C., Meyer U., Kübler N.R., Handschel J. (2008). Osseointegration of Zirconia Implants Compared with Titanium: An in Vivo Study. Head Face Med..

[15] Hashim D., Cionca N., Courvoisier D.S., Mombelli A. (2016). A Systematic Review of the Clinical Survival of Zirconia Implants. Clin. Oral Investig..

[16] Cionca N., Hashim D., Mombelli A. (2017). Zirconia Dental Implants: Where Are We Now, and Where Are We Heading?. Periodontology 2000.

[17] Chai W.L., Brook I.M., Palmquist A., van Noort R., Moharamzadeh K. (2012). The Biological Seal of the Implant-Soft Tissue Interface Evaluated in a Tissue-Engineered Oral Mucosal Model. J. R. Soc. Interface.

[18] Turkyilmaz I., Soganci G., Turkyilmaz I. (2015). Rationale for Dental Implants. Current Concepts in Dental Implantology.

[19] Gaviria L., Salcido J.P., Guda T., Ong J.L. (2014). Current Trends in Dental Implants. J. Korean Assoc. Oral Maxillofac. Surg..

[20] Chrcanovic B.R., Kisch J., Albrektsson T., Wennerberg A. (2016). Factors Influencing Early Dental Implant Failures. J. Dent. Res..

[21] Sakka S., Baroudi K., Nassani M.Z. (2012). Factors Associated with Early and Late Failure of Dental Implants. J. Investig. Clin. Dent..

[22] Quirynen M., De Soete M., van Steenberghe D. (2002). Infectious Risks for Oral Implants: A Review of the Literature. Clin. Oral Implants Res..

[23] Pye A.D., Lockhart D.E.A., Dawson M.P., Murray C.A., Smith A.J. (2009). A Review of Dental Implants and Infection. J. Hosp. Infect..

[24] Mouhyi J., Dohan Ehrenfest D.M., Albrektsson T. (2012). The Peri-Implantitis: Implant Surfaces, Microstructure, and Physicochemical Aspects. Clin. Implant Dent. Relat. Res..

[25] Scarano A., Piattelli M., Caputi S., Favero G.A., Piattelli A. (2004). Bacterial Adhesion on Commercially Pure Titanium and Zirconium Oxide Disks: An In Vivo Human Study. J. Periodontol..

[26] Rimondini L., Cerroni L., Carrassi A., Torricelli P. (2002). Bacterial Colonization of Zirconia Ceramic Surfaces: An In Vitro and In Vivo Study. Int. J. Oral Maxillofac. Implants.

[27] Roehling S., Astasov-Frauenhoffer M., Hauser-Gerspach I., Braissant O., Woelfler H., Waltimo T., Kniha H., Gahlert M. (2016). In Vitro Biofilm Formation On Titanium And Zirconia Implant Surfaces. J. Periodontol..

[28] Lee B.-C., Jung G.-Y., Kim D.-J., Han J.-S. (2011). Initial Bacterial Adhesion on Resin, Titanium and Zirconia in Vitro. J. Adv. Prosthodont..

[29] De Oliveira G.R., Pozzer L., Cavalieri-Pereira L., de Moraes P.H., Olate S., de Albergaría Barbosa J.R. (2012). Bacterial Adhesion and Colonization Differences between Zirconia and Titanium implant Abutments: An In Vivo Human Study. J. Periodontal Implant Sci..

[30] Gindri I.M., Siddiqui D.A., Frizzo C.P., Martins M.A.P., Rodrigues D.C. (2015). Ionic Liquid Coatings for Titanium Surfaces: Effect of IL Structure on Coating Profile. ACS Appl. Mater. Interfaces.

[31] Gindri I.M., Siddiqui D.A., Bhardwaj P., Rodriguez L.C., Palmer K.L., Frizzo C.P., Martins M.A.P., Rodrigues D.C. (2014). Dicationic Imidazolium-Based Ionic Liquids: A New Strategy for Non-Toxic and Antimicrobial Materials. RSC Adv..

[32] Gindri I.M., Palmer K.L., Siddiqui D.A., Aghyarian S., Frizzo C.P., Martins M.A.P., Rodrigues D.C. (2016). Evaluation of Mammalian and Bacterial Cell Activity on Titanium Surface Coated with Dicationic Imidazolium-Based Ionic Liquids. RSC Adv..

[33] Siddiqui D.A., Gindri I.M., Rodrigues D.C. (2016). Corrosion and Wear Performance of Titanium and Cobalt Chromium Molybdenum Alloys Coated with Dicationic Imidazolium-Based Ionic Liquids. J. Bio-Tribo-Corros..

[34] Gindri I.M., Siddiqui D.A., Frizzo C.P., Martins M.A.P., Rodrigues D.C. (2016). Improvement of Tribological and Anti-Corrosive Performance of Titanium Surfaces Coated with Dicationic Imidazolium-Based Ionic Liquids. RSC Adv..

[35] Cremer T., Stark M., Deyko A., Steinrück H.-P., Maier F. (2011). Liquid/Solid Interface of Ultrathin Ionic Liquid Films: [C1C1Im][Tf2N] and [C8C1Im][Tf2N] on Au(111). Langmuir.

[36] Cremer T., Wibmer L., Calderón S.K., Deyko A., Maier F., Steinrück H.-P. (2012). Interfaces of Ionic Liquids and Transition Metal Surfaces—Adsorption, Growth, and Thermal Reactions of Ultrathin [C1C1Im][Tf2N] Films on Metallic and Oxidised Ni(111) Surfaces. Phys. Chem. Chem. Phys..

[37] Villar-Garcia I.J., Smith E.F., Taylor A.W., Qiu F., Lovelock K.R.J., Jones R.G., Licence P. (2011). Charging of Ionic Liquid Surfaces under X-Ray Irradiation: The Measurement of Absolute Binding Energies by XPS. Phys. Chem. Chem. Phys..

[38] Hurisso B.B., Lovelock K.R.J., Licence P. (2011). Amino Acid-Based Ionic Liquids: Using XPS to Probe the Electronic Environment via Binding Energies. Phys. Chem. Chem. Phys..

[39] Dupin J.-C., Gonbeau D., Vinatier P., Levasseur A. (2000). Systematic XPS Studies of Metal Oxides, Hydroxides and Peroxides. Phys. Chem. Chem. Phys..

[40] Hao L., Lawrence J., Chian K.S., Low D.K.Y., Lim G.C., Zheng H.Y. (2004). The Formation of a Hydroxyl Bond and the Effects Thereof on Bone-like Apatite Formation on a Magnesia Partially Stabilized Zirconia (MgO-PSZ) Bioceramic Following CO_2_ Laser Irradiation. J. Mater. Sci. Mater. Med..

[41] Kerber S.J., Bruckner J.J., Wozniak K., Seal S., Hardcastle S., Barr T.L. (1996). The Nature of Hydrogen in X-Ray Photoelectron Spectroscopy: General Patterns from Hydroxides to Hydrogen Bonding. J. Vac. Sci. Technol. A.

[42] Schmidt M. (2001). X-ray Photoelectron Spectroscopy Studies on Adsorption of Amino Acids from Aqueous Solutions onto Oxidised Titanium Surfaces. Arch. Orthop. Trauma Surg..

[43] Bolis V., Cerrato G., Magnacca G., Morterra C. (1998). Surface Acidity of Metal Oxides. Combined Microcalorimetric and IR-Spectroscopic Studies of Variously Dehydrated Systems. Thermochim. Acta.

[44] Budyak I.L., Zhuravleva A., Gierasch L.M. (2013). The Role of Aromatic—Aromatic Interactions in Strand–Strand Stabilization of β-Sheets. J. Mol. Biol..

[45] Egawa M., Miura T., Kato T., Saito A., Yoshinari M. (2013). In Vitro Adherence of Periodontopathic Bacteria to Zirconia and Titanium Surfaces. Dent. Mater. J..

[46] Winter W., Klein D., Karl M. (2013). Micromotion of Dental Implants: Basic Mechanical Considerations. J. Med. Eng..

[47] Mathew M.T., Abbey S., Hallab N.J., Hall D.J., Sukotjo C., Wimmer M.A. (2012). Influence of pH on the Tribocorrosion Behavior of CpTi in the Oral Environment: Synergistic Interactions of Wear and Corrosion. J. Biomed. Mater. Res. Part B Appl. Biomater..

[48] Zhou F., Liang Y., Liu W. (2009). Ionic Liquid Lubricants: Designed Chemistry for Engineering Applications. Chem. Soc. Rev..

[49] Minami I. (2009). Ionic Liquids in Tribology. Molecules.

