# Supplementary material for: Dicationic Imidazolium-Based Ionic Liquid Coatings on Zirconia Surfaces: Physico-Chemical and Biological Characterization
Source: J Funct Biomater. 2017 Dec 13;8(4):50. doi: 10.3390/jfb8040050 (PMC5748557; doi:10.3390/jfb8040050)
Supplement: Supplementary file 1 [file jfb-08-00050-s001.pdf]

Supporting Information

DICATIONIC IMIDAZOLIUM-BASED IONIC LIQUID COATINGS ON ZIRCONIA SURFACES: PHYSICOCHEMICAL AND BIOLOGICAL CHARACTERIZATION

Pavan P.K. Sandhu[1], Izabelle M. Gindri[1], Danyal A. Siddiqui[1], Danieli C. Rodrigues[1]

[1]Department of Bioengineering, University of Texas at Dallas, Richardson, Texas 75080, United States

**Figure S1.**	Elemental S 2p spectra for pure IL2 (purple) and IL2-coated zirconia (green).
**Figure S2.**	Coefficient of friction of control and IL-coated zirconia under a load of 10 N.
**Figure S3.**	The modified pin-on-disk apparatus used for this study. A semi-circular fluid holder containing saliva completely submerses the stainless steel ball while the zirconia specimen is mounted to the upper fixture.

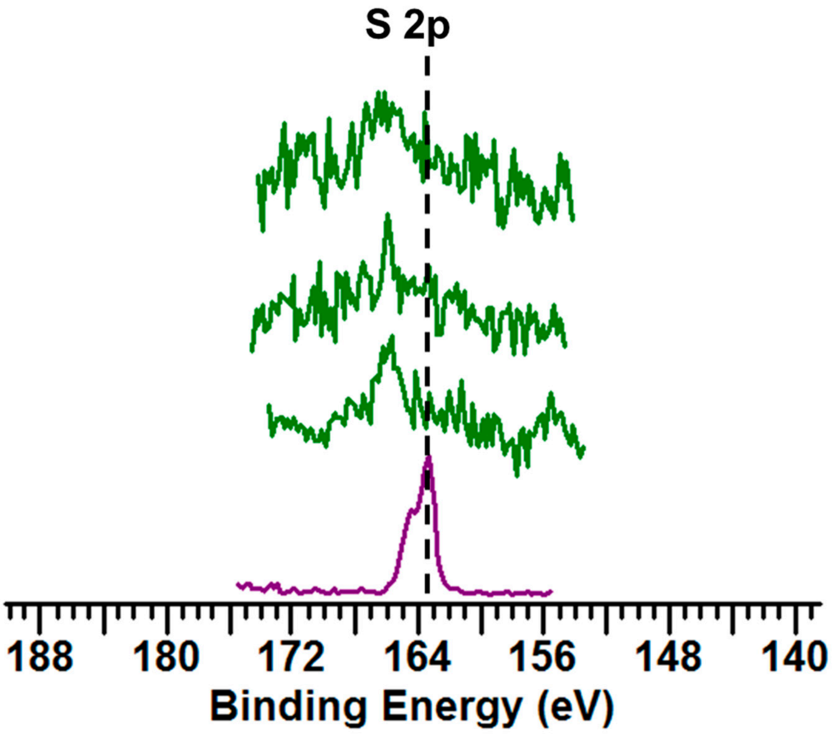

Figure S1. Elemental S 2p spectra for pure IL2 (purple) and IL2-coated zirconia (green).

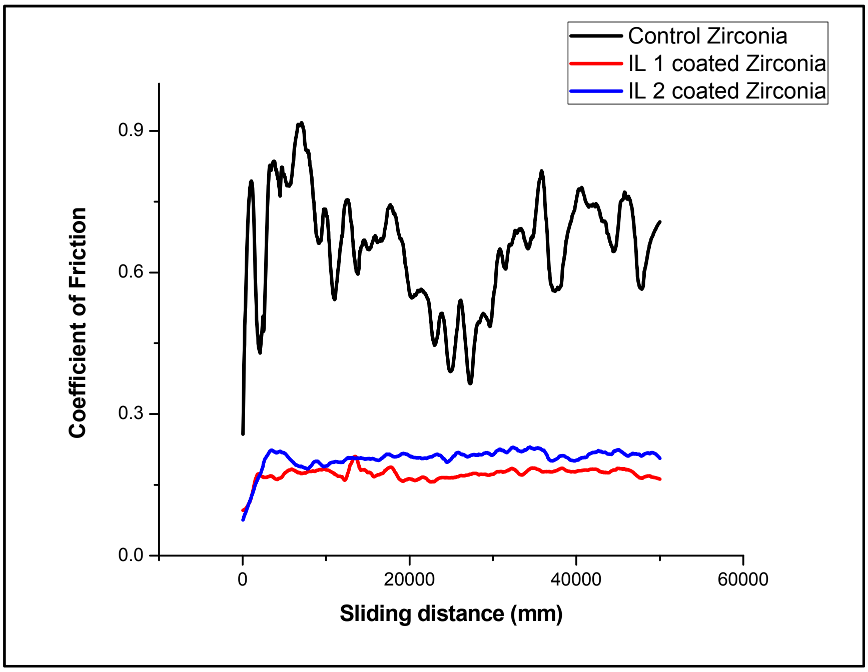

Figure S2. Coefficient of friction of control and IL-coated zirconia under a load of 10 N.

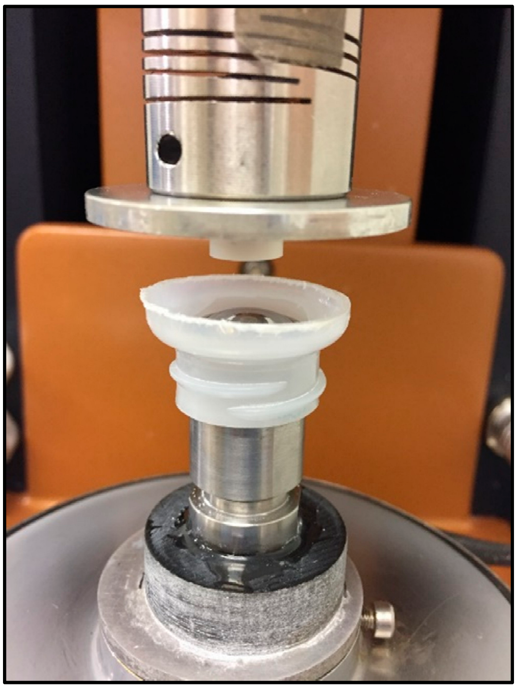

Figure S3. The modified pin-on-disk apparatus used for this study. A semi-circular fluid holder containing saliva completely submerses the stainless steel ball while the zirconia specimen is mounted to the upper fixture.
